# Reality Status Judgments of Real and Fantastical Events in Children’s Prefrontal Cortex: An fNIRS Study

**DOI:** 10.3389/fnhum.2019.00444

**Published:** 2019-12-20

**Authors:** Hui Li, Tao Liu, Jacqueline D. Woolley, Peng Zhang

**Affiliations:** ^1^School of Education, Central China Normal University, Wuhan, China; ^2^School of Management, Zhejiang University, Hangzhou, China; ^3^Department of Psychology, The University of Texas at Austin, Austin, TX, United States; ^4^Faculty of Psychology and Behavior, Tianjin Normal University, Tianjin, China

**Keywords:** reality judgment, fantasy, child development, fNIRS, PFC

## Abstract

The present study aimed to examine neural mechanisms underlying the ability to differentiate reality from fantasy. Using functional near-infrared spectroscopy (fNIRS), we measured prefrontal activations in children and adults while they performed a reality judgment task. Participants’ task was to judge the reality status of events in fantastical and realistic videos. Behavioral data revealed that, although there was no accuracy difference, children showed significantly longer reaction times in making the judgments than did adults. The fNIRS data consistently revealed higher prefrontal activations in children than in adults when watching the videos and judging the reality of the events. These results suggest that when making judgments of event reality, children may require more cognitive resources and also mainly rely on their own personal experiences.

## Introduction

Television, movie, and video viewing have become a staple of children’s daily lives. Two-thirds of children under age 7 in the United States watch television every day, usually for around 2 h (Rideout and Hamel, 2006). Similar patterns are found in other countries; for example, in China, 93% of Chinese preschoolers watch television every day for about an hour and half (Li et al., 2014). Young children are also increasingly becoming Internet consumers; video sharing sites are one of the first sites that young children visit on the Internet, and these sites are especially popular with younger children (Holloway et al., 2013).

Children’s television, movies, and videos often contain a mix of real and fantastical events. For example, “Elmo” teaches children about what it means to be alive, and “Dora the Explorer” teaches children about animals while playing with a magic stick. In addition, animated cartoons can easily violate the laws of nature; characters can appear and disappear, or turn into something else entirely. This raises the important question of how we ensure that children learn what we expect them to learn from this arguably confusing content (Hopkins and Weisberg, 2017). An inability to distinguish real from not-real could limit the amount and type of real-world learning that takes place, and could also potentially generate misconceptions.

What do we know about children’s ability to judge the reality status of real and fantastical events in videos? More generally, the ability to differentiate reality from fantasy emerges early in development, with children beginning to use words like “real” and “pretend” by age 2 (Woolley and Wellman, 1990). Research by Flavell et al. (1990) indicates that 3-year-old children do not always understand that television is representational, and sometimes think that real objects are actually inside the television. Goldstein and Bloom (2015) observed a similar pattern – preschool-age children judged actors on videos to really be experiencing the emotions they were portraying. Yet other studies show the reverse pattern – children incorrectly judge video and television content to be unreal. Wright et al. (1994) observed a bias in children aged 5 and younger to incorrectly assume that all television content was fictional. Work by Li et al. (2015) similarly indicates that young 4-year-olds often underestimate the reality status of real events in videos. They showed children real and fantastical events and asked them to judge their reality status. Although children performed well when asked to judge the reality of fantastical events, they often claimed that real events could not actually happen in real life. Li et al. suggest that, in judging the reality of events on television and video, children rely heavily on their personal experience.

Although previous behavioral studies have consistently documented these sorts of errors in young children, the neural correlates underlying them are still not understood. The goal of the present research is thus to investigate the neural underpinnings of children’s ability to make the fantasy-reality distinction for video content, and to determine whether the same neural structures are involved for both children and adults. Research by Abraham et al. (2008) found selective activations of the anterior prefrontal cortex (PFC) and the precuneus/posterior cingulate when adult participants evaluated whether it was possible to interact with real people vs. with fictional characters. They suggest that real people activate more autobiographical memory retrieval, and propose that whether or not character-type information is coded in self-relevant terms is a key factor in differentiating reality from non-reality. Altmann et al. (2014) examined the neurocognitive processes when adults read purportedly real or invented narratives; results indicated that participants had faster reaction times (RTs) when a story was believed to be real vs. fictional. In addition, fMRI data indicated that the factual and the fictional contexts reflected different levels of simulation: they propose that reading factual texts mostly involves thinking about actions and their outcomes, as reflected in increased activations mainly in social-brain areas such as the temporo-parietal junction (TPJ), whereas reading fiction normally increases activation in cognitive control areas such as the dorsolateral prefrontal cortex (dlPFC).

Woolley (1997) argues that children are not “fundamentally different” from adults in their ability to distinguish fantasy and reality. Han et al. (2007) provide initial evidence that the underlying mental processes may differ. Using fMRI, Han et al. (2007) recorded 10-year-old children’s activations while viewing both silent movies showing humans in real-life situations and cartoon clips with non-human characters. The results showed that children’s medial prefrontal cortex (mPFC) was activated when watching both real and cartoon characters. In contrast, previous research with adults had revealed that the mPFC was only activated in processing the mental activity of real characters in movie clips (Han et al., 2005). The authors suggest that adults respond differently to real and fictional worlds – automatically attributing mental states to characters in the former but not the latter, whereas children do not.

Due to the technical limitations of fMRI, it is difficult to examine brain activation in younger children, who cannot keep their body motionless for long periods of time. Functional near-infrared spectroscopy (fNIRS) is non-invasive brain imaging technique that has relatively high temporal resolution, places few physical constraints, and is tolerant to motion artifact and electromagnetic noise (for review, see Scholkmann et al., 2014). Therefore, it is ideal for use with young children (Perlman et al., 2014; Liu et al., 2017). One recent study with fNIRS revealed that watching fantastical events performed by cartoon characters leads to increased activation in children’s dlPFC relative to playing with them using a touch screen (Li et al., 2018). This study established the feasibility of using this method to address children’s fantasy-reality distinction; however, the neural correlates underlying the ability to discriminate fantasy from reality in children remain largely unknown. To address this question, the present study used fNIRS to examine activation patterns in the PFC of children and adults while they performed a real-fantastical judgment task.

## Materials and Methods

Thirty-five participants, 16 children (78.54 ± 7.40 months, six girls) and 19 adults (257.53 ± 28.94 months, 10 women) viewed videos varying by character (real vs. fantastical) and event (real vs. fantastical) type in a mixed design, forming four conditions, i.e., fantastical character fantastical event (FF), fantastical character real event (FR), real character fantastical event (RF), and real character real event (RR). Three children’s behavioral data were missing due to mis-manipulation of the presentation software, leaving 32 participants in the final data analyses. All children’s parents signed informed consent forms and were paid 200 RMB (30.8 USD) for their participation. This study was approved by the Institutional Review Board of Central China Normal University.

**FIGURE 1:**
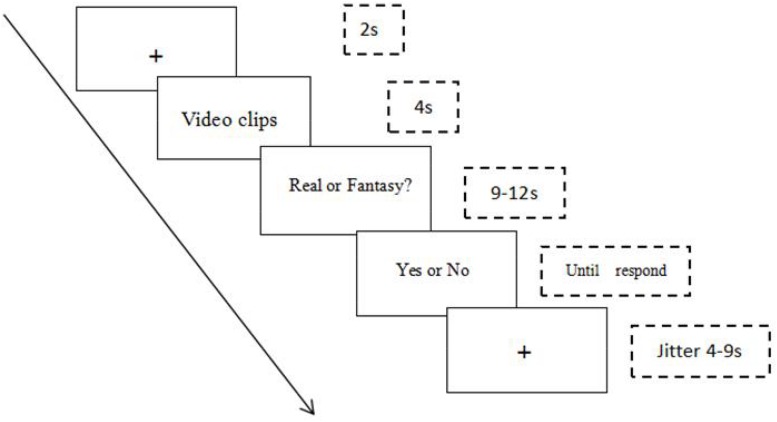
Procedure for a reality judgment trial.

Each participant was shown 40 short video clips, each of which portrayed a central event lasting about 4000 ms. All the clips were taken from the popular cartoons *SpongeBob* and *Happy Satellite* (see Appendix A for the list of clip descriptions) (Skolnick and Bloom, 2006). Videos were displayed in the pre-determined random order indicated in Appendix A across 40 trials. Each video clip was followed by one question. Participants’ task was to judge the reality of the events (e.g., “Do you think it is possible for two people to take a boat ride in real life?”) by pressing “1” on a keypad for “Yes” and “0” for “No” (see Figure 1).

Each trial began with a fixation cross (duration: 2000 ms), which was followed by a single video clip for 4000 ms. Each video clip was accompanied by a taped verbal description of the event. After each event, a question cue (a ringing sound) was presented to ready the participant for the test question. Variable jitter times were inserted before the video clips (4000–9000 ms) to help maintaining participants’ attention on the task. Prior to and after all trials, participants were respectively given 30 s to rest.

**FIGURE 2:**
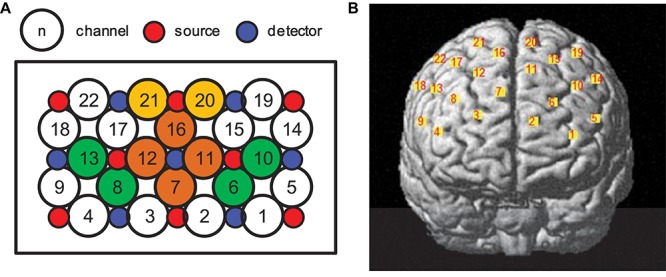
**(A)** Channel arrangement, and **(B)** positions of fNIRS channels. Circles in orange covered the medial part of prefrontal cortex (mPFC), circles in green covered the left and right prefrontal cortex (lPFC and rPFC), circles in yellow covered the dorsolateral prefrontal cortex (dlPFC).

A multichannel fNIRS system (LABNIRS; Shimadzu, Japan) was used to record hemodynamic changes in the prefrontal cortices covered by 22 channels (see Figure 2). The bottom channels covered the Fp1–Fp2 line and the bottom central probe covered the Fpz point according to the international 10–10 system (Vespignani and Braun, 2009). We mainly analyzed oxygenated hemoglobin (HbO), since it is the most sensitive parameter of regional cerebral blood flow and provides a robust correlation with the BOLD signal (Hoshi et al., 2001; Huppert et al., 2006). The duration for each trial ranged approximately from 10 to 17 s, that is, 0.06–0.1 Hz. To remove global trends and systematic noise such as heartbeats and breath, and to include more task-related cortical activations, the raw HbO data were preprocessed by a band-pass filter (0.01–0.1 Hz). A baseline correction was then conducted by subtracting the mean value of the last 15 s resting period prior the first trial from the filtered HbO data. Since the fNIRS measures relative but not absolute hemodynamic concentrations like fMRI, the HbO data was further converted into *z* scores using the mean value and standard deviation from the same 15 s baseline period (Matsuda and Hiraki, 2006). To increase signal-to-noise ratio, the HbO data belonging to the same region of interest (ROI) were averaged. Finally, group-averaged data were obtained across all trials for both the video watching and reality judgment periods.

Positions of the fNIRS channels were measured using a 3D electromagnetic tracking device (FASTRAK; Polhemus, Colchester, VT, United States), and then a probabilistic registration method was used to estimate each channel’s corresponding position in the Montreal Neurological Institute (MNI) space via NIRS-SPM. Because the MNI space was established for adults but not children, the estimated channel positions may be not accurate. To avoid potential deviations in children, we intentionally neglected the peripheral channels and also the ones with low registration probabilities (i.e., <0.60). Table 1 shows positions of all fNIRS channels. Finally, we focused on four ROIs, that is, the mPFC (Ch 7, 11, 12, and 16), the left prefrontal cortex (lPFC; Ch 6 and 10), the right prefrontal cortex (rPFC; Ch 8 and 13), and the dlPFC (Ch 20 and 21).

**TABLE 1:** Positions of all fNIRS channels.

**Channel**	**Anatomical label**	**Percentage of Overlap**
CH01	11 – Orbitofrontal area	0.50
CH02	10 – Frontopolar area	0.70
CH03	10 – Frontopolar area	0.75
CH04	11 – Orbitofrontal area	0.57
CH05	10 – Frontopolar area	0.57
CH06	10 – Frontopolar area^∗^	0.96
CH07	10 – Frontopolar area^∗^	1.00
CH08	10 – Frontopolar area^∗^	0.94
CH09	10 – Frontopolar area	0.57
CH10	10 – Frontopolar area^∗^	0.69
CH11	10 – Frontopolar area^∗^	1.00
CH12	10 – Frontopolar area^∗^	1.00
CH13	10 – Frontopolar area^∗^	0.64
CH14	46 – Dorsolateral prefrontal cortex	0.86
CH15	10 – Frontopolar area	0.59
CH16	10 – Frontopolar area^∗^	0.78
CH17	10 – Frontopolar area	0.55
CH18	46 – Dorsolateral prefrontal cortex	0.76
CH19	9 – Dorsolateral prefrontal cortex	0.56
CH20	9 – Dorsolateral prefrontal cortex^∗^	0.88
CH21	9 – Dorsolateral prefrontal cortex^∗^	0.90
CH22	9 – Dorsolateral prefrontal cortex	0.55

*^∗^Indicates region of interest in the present study.*

## Results

To examine differences between children and adults in their judgments of event reality, we conducted mixed ANOVAs (Age [2] × Character [2] × Event [2]) on the behavioral and fNIRS data independently. The statistical analyses were conducted using the Statistical Package for the Social Sciences 19 (SPSS), and the significance level was set at *p* < 0.05. To control for false positives, all *p*-values were corrected by false discovery rate (FDR = 0.05).

### Behavioral Data

We calculated RT and accuracy as performance indices. Concerning RT, there was a significant main effect of Age [*F*(1,30) = 19.89, *p* < 0.001, η_p_^2^ = 0.40], with longer RT in children (*M* ± *SE*: 1953.34 ± 190.65 ms) than in adults (849.78 ± 150.70 ms). Although the main effect of Character was not significant [*F*(1,30) = 3.29, *p* = 0.08, η_p_^2^ = 0.10], participants tended to show longer RT on real (1511.39 ± 161.43 ms) than on fantastical (1197.68 ± 80.85 ms) characters. There was also a significant main effect of Event [*F*(1,30) = 9.85, *p* < 0.01, η_p_^2^ = 0.25], with longer RT on real (1607.45 ± 174.45 ms) than on fantastical (1195.66 ± 93.71 ms) events.

There was also a significant interaction between Age and Character [*F*(1,30) = 3.62, *p* = 0.06, η_p_^2^ = 0.11]. Simple effects tests and multiple comparisons revealed that for real characters, children’s RT (2235.25 ± 257.69 ms) was longer than adults’ (843.03 ± 213.16 ms) [*F*(1,30) = 17.33, *p* < 0.001, η_p_^2^ = 0.37], and for fantastical characters, children’s RT (1671.44 ± 183.43 ms) was also longer than adults’ (856.52 ± 151.72 ms) [*F*(1,30) = 11.72, *p* < 0.001, η_p_^2^ = 0.28]. In addition, children’s RT was longer when events were performed by real than by fantastical characters [*F*(1,30) = 5.81, *p* < 0.05, η_p_^2^ = 0.16].

**FIGURE 3:**
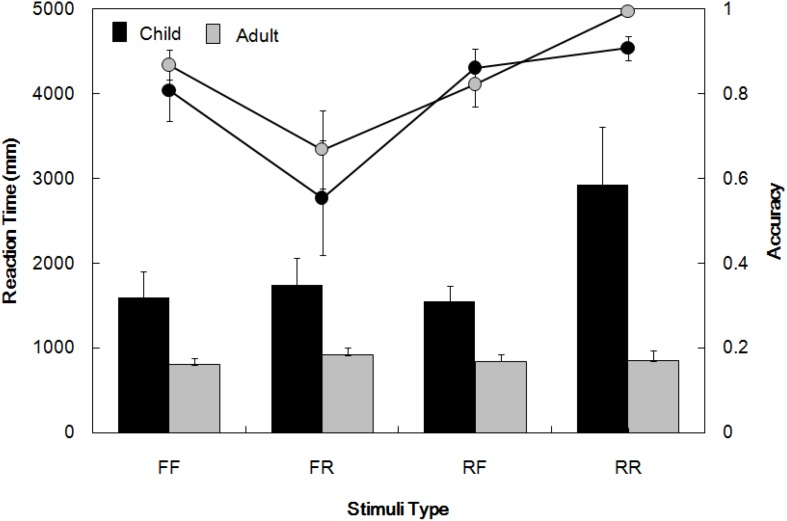
Behavioral performance yielded by children and adults. FF, FR, RF, and RR represent fantastical character with fantastical event, fantastical character with real event, real character with fantastical event, and real character with real event. The bar graphs represent reaction time, while the line graphs represent accuracy. Bars indicate standard errors.

The analyses also revealed a significant interaction between Age and Event [*F*(1,30) = 7.24, *p* < 0.05, η_p_^2^ = 0.19]. Simple effects tests and multiple comparisons (Bonferroni correction) revealed that for real events, children’s RT (2335.76 ± 268.85 ms) was longer than adults’ (879.15 ± 222.39 ms) [*F*(1,30) = 17.43, *p* < 0.001, η_p_^2^ = 0.37], and for fantastical events, children’s RT (1570.93 ± 144.42 ms) was also longer than adults’ (820.40 ± 119.46 ms) [*F*(1,30) = 16.04, *p* < 0.001, η_p_^2^ = 0.35]. In addition, children’s RT was longer in judging the reality status of real than fantastical events [*F*(1,30) = 14.31, *p* < 0.001, η_p_^2^ = 0.32], whereas no RT differences were revealed between judgments of real and fantastical events in adults. No RT differences were observed between judgments involving real and fantastical characters in adults. There were no significant differences between children and adults in accuracy. Figure 3 illustrates the behavioral results of RT and accuracy.

Taken together, children consistently showed longer RT than adults. Furthermore, children needed more time to make a decision when faced with real events and characters relative to when they judged fictional events and characters, whereas adults spent equivalent amounts of time judging both.

### fNIRS Data

#### Intra-Brain Activation

**FIGURE 4:**
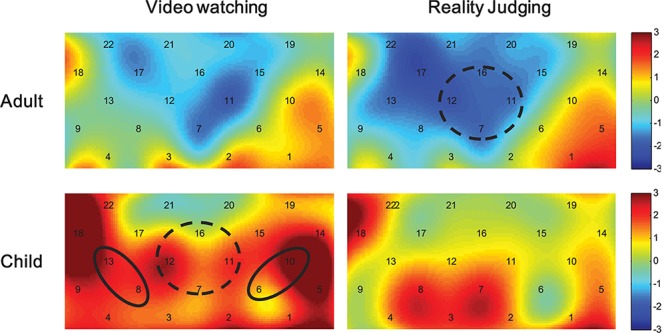
Heap maps of *t* values comparing activations during the video watching and reality judgment periods to the resting baseline in both the child and adult participants. Solid circles indicate the ROIs that showed significantly increased activations (*p* < 0.05 FDR corrected), whereas the dashed circles indicate *p* < 0.05 uncorrected.

We first examined the cortical activations involved in the judgment task, and applied one-sample *t*-tests on preprocessed HbO data during the video watching and reality judgment periods for both the adult and child participants. Figure 4 shows heat maps of the *t* values. For adults, no ROI areas were activated compared with the resting baseline (*p*s > 0.111) while watching videos, but there was a tendency to show decreased activations in the mPFC [-1.15 ± 0.56; *t*(18) = -2.183, *p* = 0.043, uncorrected and not significant after FDR correction] while judging the reality of the events (*p*s > 0.173 for the other ROIs).

By contrast, for children, when watching the videos they showed increased activations in the lPFC [0.96 ± 0.32; *t*(18) = 3.525, *p* = 0.003, FDR corrected], the rPFC [1.38 ± 0.60; *t*(18) = 2.561, *p* = 0.022, FDR corrected], and also showed the same tendency in the mPFC [0.76 ± 0.45; *t*(18) = 1.941, *p* = 0.071, uncorrected]. No ROI areas were significantly activated when judging the reality of the events in children (*p*s > 0.095).

In addition, we also analyzed the Deoxy data to verify the results found by the Oxy data. The Deoxy data were preprocessed in the same way as the Oxy data, and the *t*-test revealed significantly decreased activations compared with the resting baseline regardless of watching or judging periods in adults (*p*s < 0.005, FDR corrected). The children also showed the same trend of decreasing of prefrontal activations, especially in the dlPFC, the lPFC and the rPFC during the video watching period (*p*s < 0.05, FDR corrected; *p* = 0.099 in mPFC uncorrected), and in the left and right PFC during the reality judgment period (*p*s < 0.05, FDR corrected).

#### Activation Differences

We then compared the activation differences between the adult and child participants using the same Age [2] × Character [2] × Event [2] ANOVA. While watching the event videos, the analysis revealed significant main effects of Age in the mPFC [*F*(1,33) = 6.516, *p* < 0.05, η_p_^2^ = 0.17] and the rPFC [*F*(1,33) = 4.573, *p* < 0.05, η_p_^2^ = 0.12], and also an Age by Character interaction in the rPFC [*F*(1,33) = 5.112, *p* < 0.05, η_p_^2^ = 0.13]. No other main effects and interactions were demonstrated. Simple effects tests and multiple comparisons (Bonferroni correction) revealed that children showed higher activations than adults in the mPFC. In the rPFC, the same trend of increased activation in children compared with adults was revealed, especially while watching events performed by fantastical characters (*p* < 0.05; *p* = 0.080 while watching real characters).

**FIGURE 5:**
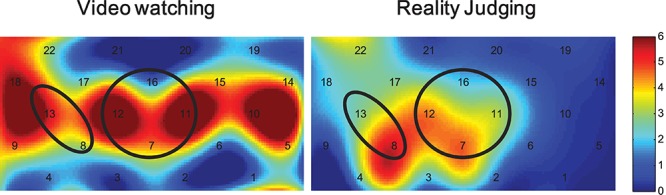
Heap maps of *F* values for the main effect of Age (child vs. adult) during the video watching and reality judgment periods. Solid circles indicate the ROIs that showed significantly different activations (*p* < 0.05 FDR corrected).

When judging the reality of events, the ANOVA consistently revealed significant main effects of Age in the mPFC [*F*(1,33) = 6.128, *p* < 0.05, η_p_^2^ = 0.17] and the rPFC [*F*(1,33) = 4.650, *p* < 0.05, η_p_^2^ = 0.12] and a main effect of Character in the mPFC [*F*(1,33) = 7.700, *p* < 0.01, η_p_^2^ = 0.19]. The Age by Event interaction was also significant in the rPFC [*F*(1,33) = 5.728, *p* < 0.05, η_p_^2^ = 0.15]. No other significant main effects or interactions were revealed. Simple effects tests and multiple comparisons (Bonferroni correction) revealed higher mPFC activations in the children compared with the adults (*p* < 0.05). In addition, the mPFC also showed higher activations while judging the events performed by real characters than those performed by fantastical characters (*p* < 0.01). Importantly, the children tended to show higher rPFC activations when judging the real events than the fantastical events (*p* = 0.055), and the rPFC activation differences between children and adults were mainly revealed when judging the real events (*p* < 0.05). Figure 5 shows heap maps of *F* values for the main effects of Age (child vs. adult) during the video watching and reality judgment periods.

The same mixed three-way ANOVA revealed no significant main effects and interactions in both the video watching (*p*s > 0.099) and reality judgment periods (*p*s > 0.080), except for a main effect of Age in the mPFC when judging the reality of events [*F*(1,33) = 7.185, *p* < 0.05, η_p_^2^ = 0.18]. During the video watching and reality judgment periods, children tended to showed decreased Deoxy but increased Oxy, confirming that the present findings revealed by the Oxy data resulted from cognitive processing involved in the reality judgment task.

#### Behavioral-Activation Relation

We then directly examined relations between participants’ behavioral performance and their prefrontal activations. There was no significant relation between the prefrontal activations and RT while judging the reality of the events in children or adults (*p*s > 0.10). Concerning the ACC, children tended to show a positive correlation between their judgment accuracy on RR events and the mPFC activation in the reality judging period (*r* = 0.544, *p* = 0.054). For adults, the same trend was revealed between their mPFC activation and both the general judgment accuracy (watching period: *r* = 0.443, *p* = 0.057; judging period: *r* = 0.411, *p* = 0.081) and accuracy on FR events (watching period: *r* = 0.488, *p* = 0.034; judging period: *r* = 0.497, *p* = 0.030).

## Discussion

The present study aimed to examine neural differences between children and adults in judging the reality of an event. To achieve this goal, we measured both children’s and adults’ prefrontal activations using fNIRS in an event-reality-judgment task. The present study provides preliminary evidence regarding the neural structures involved for both children and adults in making reality judgments, thus may contribute to the literature on the development of an understanding of reality and fantasy.

The main findings of the present results are two-fold. First, the behavioral data suggest that children required more cognitive resources (Heekeren et al., 2004; Yang et al., 2009) to judge the real events with real characters than to judge all the other events. Adults, on the other hand, needed significantly fewer resources to judge all events, and used equal amounts of cognitive resources across all four types of events.

Second, consistent with behavioral data, the fNIRS data also revealed higher prefrontal activations in children than in adults while watching and judging the reality of events, especially in the mPFC and rPFC. More importantly, when judging the real events or the events performed by real characters, the children showed higher prefrontal activations, confirming the behavioral findings. In addition, positive correlations were revealed between children’s mPFC activations and their judgment accuracy on RR events. The medial part of PFC has been indicated in self referential thinking and autobiographical memory retrieval (Ramnani and Owen, 2004; Cavanna and Trimble, 2006; Gilbert et al., 2006; Svoboda et al., 2006), and is selectively engaged when processing contexts containing real entities (Abraham and von Cramon, 2009). Thus, children may mainly rely on their self-referential experience to judge the reality of events. This finding is consistent with Han et al.’s (2007) research showing that adults’ mPFC was not activated during viewing cartoon clips with fantastical characters, whereas children’s mPFC was activated when viewing events with both real and fantastical characters.

In addition, the left anterior of PFC is closely associated with verbal working memory (Hartley et al., 2000), memory retrieval (Wolf et al., 2006) and mental simulations (Altmann et al., 2014). Thus, in making judgments of real events, it appears that children may need additional cognitive resources from memory (Liu et al., 2012). Taken together, these results may support theoretical claims made by Woolley and Ghossainy (2013) that children rely on personal experience in making reality status judgments.

The present study focuses only on the PFC. Future research should explore more social-brain areas, such as the inferior frontal gyrus and the temporal parietal junction, since reality judgment involves aspects of social cognition, and the mirror neuron system connects one’s own experience with social stimuli yielded by other persons. Additionally, Shtulman and Carey (2007) suggest that children and adults may use different criteria to distinguish between possible and impossible events. Exploring the neural bases of children’s and adults’ understanding of possible, impossible, and improbable events in the media would inform this hypothesis, and thus is an additional important topic for future research. Third, the present study only recruited children between 78.54 ± 7.40 months of age; more younger and older children should be examined in future studies to confirm the present findings.

## Data Availability Statement

The datasets generated for this study are available on request to the corresponding author.

## Ethics Statement

The studies involving human participants were reviewed and approved by the Institutional Review Board of Central China Normal University. Written informed consent to participate in this study was provided by the participants’ legal guardian/next of kin.

## Author Contributions

HL and JW: conceptualization. TL and PZ: methodology. HL, TL, and JW: writing original draft and review and editing.

## Conflict of Interest

The authors declare that the research was conducted in the absence of any commercial or financial relationships that could be construed as a potential conflict of interest.

## Appendix A

**Table:** List of Stimulus Item Descriptions.

**Categorization**	**Real character**	**Fantastical character**
Real event	4. A boy and a girl are talking as they walk.	3. SpongeBob is singing a song with his friend.
	6. Students are saying hi to their teacher.	21. SpongeBob is asking to unfold a chair.
	8. A boy is waiting for his friend to get out of the car.	23. SpongeBob is watching TV with his friend.
	10. A boy is calling his friend on the phone.	27. SpongeBob is taking a boat ride with his friend.
	11. Two children are arm wrestling.	33. SpongeBob is receiving a package.
	12. The doctor is giving the students their check-ups.	34. SpongeBob is saying hi to his friend.
	15. A teacher is teaching a class.	35. SpongeBob is crying.
	16. A boy is having dinner with his mother.	36. SpongeBob is talking on the intercom.
	28. Boys are running around on the playground.	37. SpongeBob is going to sleep.
	40. Boys are eating.	39. SpongeBob is making a hamburger.
Fantastical event	5. A boy is hiding behind a magic shield.	1. SpongeBob is going into a container that is smaller than his body.
	9. A boy is flying on a broom.	2. SpongeBob and his friend are flying out of the house.
	14. A girl is moving a man by magic.	7. SpongeBob and his friend are rotating in the sky.
	17. A boy is jumping out of the computer.	13. SpongeBob is transforming his body after absorbing water.
	18. A boy is stuck in a blast of light.	24. SpongeBob is flying like a rocket.
	19. A boy is flying to chase adults.	25. SpongeBob is running on the wall.
	20. Two boys are flying in the sky.	29. SpongeBob is putting Patrick Star into his mouth.
	22. A girl is making two men stand still with light.	31. SpongeBob is twisting his body while floating in the air.
	26. A boy made two girls appear by pressing a button.	32. SpongeBob is transforming his arms.
	30. Two adults are shaking hands to generate current.	38. SpongeBob is elongating his mouth to drink water.
